# Editorial to Advances in Minimally Invasive Spine Surgery

**DOI:** 10.3390/jcm13216569

**Published:** 2024-11-01

**Authors:** Gabriel C. Tender, George A. Crabill

**Affiliations:** Department of Neurosurgery, Louisiana State University Health Sciences Center, New Orleans, LA 70112, USA; gcrabi@lsuhsc.edu

Degenerative spinal pathology is a leading cause of morbidity in the United States with a predicted increase in prevalence as the population ages. For select patients, surgery can increase quality of life and allow patients to return to work. Traditional open surgical interventions involve relatively large incisions and extensive soft tissue disruption. As general surgery evolved to smaller incisions while achieving the same results (e.g., laparoscopic cholecystectomy), so did spine surgery.

Starting around the turn of the century, the usage of the operative microscope allowed for minimally invasive spine (MIS) surgery techniques to emerge, initially in the form of tubular microdiscectomy (MCD). This technique, employing an 18 mm diameter tubular retractor, allowed for small incisions and minimal soft tissue disruption and proved particularly advantageous in obese patients. Another observed benefit was the minimal incidence of infections. The same approach could also be used to repair pars interarticularis fractures (Contribution 5).

The next minimally invasive technique, the transforaminal lumbar interbody fusion (TLIF), was basically an expansion of the microdiscectomy technique. Surgeons noted that, using a 22 mm diameter tubular retractor, the entire medial facet and the tip of the lateral facet could be removed, allowing perfect access to Kambin’s triangle and, thus, allowing the insertion of a TLIF cage with minimal retraction or even exposure of the spinal sac [1]. This technique also allowed for contralateral decompression by removing the yellow ligament underneath the spinous process and contralateral lamina. Currently, a variety of cages are being developed in order to maximize the footprint in contact with the two endplates (and implicitly the fusion rates) (Contribution 3). Moreover, robotic assistance has become increasingly helpful with the MIS TLIF techniques [2].

An auxiliary technique accompanying the minimally invasive TLIF was the placement of percutaneous pedicle screws and rods [3]. This technique initially employed the usage of two C-arms (antero-posterior and lateral views), but this has since been replaced by navigation and/or robotics [4]. This technique was also used for the temporary stabilization of spinal fractures [5]. The same anatomic landmarks have also been used for the placement of Jamshidi needles for the injection of cement in osteoporotic or fractured vertebral bodies (also known as vertebroplasty or kyphoplasty) (Contribution 2). 

Another important step forward in minimally invasive spine surgery was the development of the extreme lateral transpsoas approach (XLIF or LLIF). Initially described as a two-incision approach, this technique has now been modified to a single incision (typically just above the iliac crest), but with the option of cranio-caudal extension to accommodate multilevel discectomies or corpectomies. One major drawback of this approach is the inability to address the L5-S1 level due to the unfavorable psoas and vascular anatomy. However, at L4-5 and above, this technique allows for the insertion of cages with a massive footprint as well as expansion capabilities. Currently, variations of this technique are being developed, with the patient in prone position (also known as “prone transpsoas” or PTP) [6]. 

One of the common postoperative complications of the XLIF approach was anterior psoas dysesthesia, potentially due to the genitofemoral nerve manipulation, or possibly just the psoas dissection. Therefore, a variation of the XLIF technique, called oblique lateral interbody fusion (OLIF), involved a more anterior skin incision and retraction, rather than dissection, of the psoas muscle. Currently, this technique is frequently employed to create marked focal lordosis by releasing the anterior longitudinal ligament (which is directly visualized) and inserting hyper lordotic (and often expandable) cages (also known as anterior column release, or ACR) [7]. 

While all of the above techniques typically require the use of an operative microscope, in recent years, endoscopic techniques have become more popular in the United States. These techniques appear to be the preferred choice for Asian spine surgeons, but have not been widely embraced by US surgeons yet. 

The novice surgeon typically starts with the transforaminal approach, accessing the Kambin’s triangle just outside the lateral facet (which can also be partially removed with a high speed drill or Kerrison rongeur for larger access) [8]. The 7 mm skin incision is barely visible postoperatively and, due to minimal bony work and soft tissue disruption, the patients need minimal postoperative pain medication. Moreover, some of these procedures can be carried out under local anesthesia in an awake patient (Contribution 1).

The next step in endoscopic spine surgery is performing a TLIF through the 7 or 9 mm portal [9]. This technique may require expandable cages, but the graft post-fill capabilities maintain a remarkably high fusion rate. Whether followed by unilateral or bilateral percutaneous pedicle screw/rod placement, the endoscopic TLIF patients go home the same day, making this an attractive procedure for ambulatory surgery centers (ASCs). 

Another endoscopic spine technique that requires special instruments is the interlaminar approach [10]. This is particularly useful in patients with paracentral disc herniations that may be difficult to access through the transforaminal approach (Contribution 4). In this approach, the access portal also acts as a retractor for the dura mater, making the herniation removal safe from encountering an accidental durotomy. 

Advanced endoscopic spine techniques involve cervical laminectomy for central canal decompression [11]. Similarly, special instruments are required to protect the dura mater and underlying spinal cord, but the benefits (minimal soft tissue disruption and same-day discharge) make it worth learning.

In conclusion, while minimally invasive spine surgery may have a steep learning curve, savvy patients and market demands have made it a requirement for the spine surgeon, especially in competitive environments. This Special Issue underscores the latest developments in minimally invasive spine surgery and stimulates further research and clinical applications for the practicing spine surgeon.
